# Utility of MRI in Quantifying Tissue Injury in Cervical Spondylotic Myelopathy

**DOI:** 10.3390/jcm12093337

**Published:** 2023-05-08

**Authors:** Ali Fahim Khan, Grace Haynes, Esmaeil Mohammadi, Fauziyya Muhammad, Sanaa Hameed, Zachary A. Smith

**Affiliations:** 1 Department of Neurosurgery, University of Oklahoma Health Sciences Center, Oklahoma City, OK 73104, USA; alifahimkhan@gmail.com (A.F.K.);; 2 Stephenson School of Biomedical Engineering, University of Oklahoma, Norman, OK 73019, USA

**Keywords:** cervical spondylotic myelopathy, magnetic resonance imaging, functional magnetic resonance imaging, spinal cord imaging, spinal cord injury, cervical spine, spondylosis

## Abstract

Cervical spondylotic myelopathy (CSM) is a progressive disease that worsens over time if untreated. However, the rate of progression can vary among individuals and may be influenced by various factors, such as the age of the patients, underlying conditions, and the severity and location of the spinal cord compression. Early diagnosis and prompt treatment can help slow the progression of CSM and improve symptoms. There has been an increased use of magnetic resonance imaging (MRI) methods in diagnosing and managing CSM. MRI methods provide detailed images and quantitative structural and functional data of the cervical spinal cord and brain, allowing for an accurate evaluation of the extent and location of tissue injury. This review aims to provide an understanding of the use of MRI methods in interrogating functional and structural changes in the central nervous system in CSM. Further, we identified several challenges hindering the clinical utility of these neuroimaging methods.

## 1. Introduction

Cervical spondylotic myelopathy (CSM) is one of the leading causes of cervical spinal cord (CSC) dysfunction, typically affecting adults aged 50 years or more. The prevalence of CSM is expected to rise worldwide due to the aging population [1]. It is associated with a low quality of life [2]. Tissue injuries associated with CSM include cord compression [3], demyelination of spinal tracts [4], and gray matter atrophy in the spinal cord [5]. Moreover, there is emerging evidence of tissue injury in CSM patients beyond the spinal cord, i.e., manifested in the form of structural changes [6] and functional reorganization within the brain [7]. CSM typically progresses gradually, spanning several years [8]. If untreated, permanent tissue injury may occur, leading to a wide array of motor and sensory symptoms, including debilitating pain and disability [9]. Therefore, accurate and reliable means of assessing tissue injury are essential for effective disease management.

**Figure 1:**
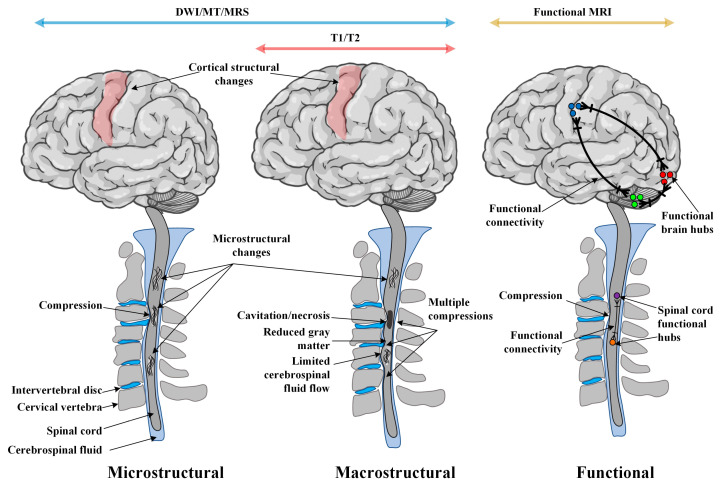
Magnetic resonance imaging (MRI) methods used in CSM research. Tissue injury in CSM patients typically occurs in the cervical spinal cord but may also be present in the brain. Such tissue injuries manifested as structural changes have been reported using different MRI methods. T1- and T2-weighted MRI contrasts can typically assess macrostructural changes, whereas DWI, MT, and MRS can also interrogate microstructural tissue changes. Beyond structural changes, functional MRI provides an opportunity to assess functional connectivity changes in the brain and the spinal cord. Abbreviations: DWI: diffusion-weighted imaging, MT: magnetization transfer, MRS: magnetic resonance spectroscopy.

**Figure 2:**
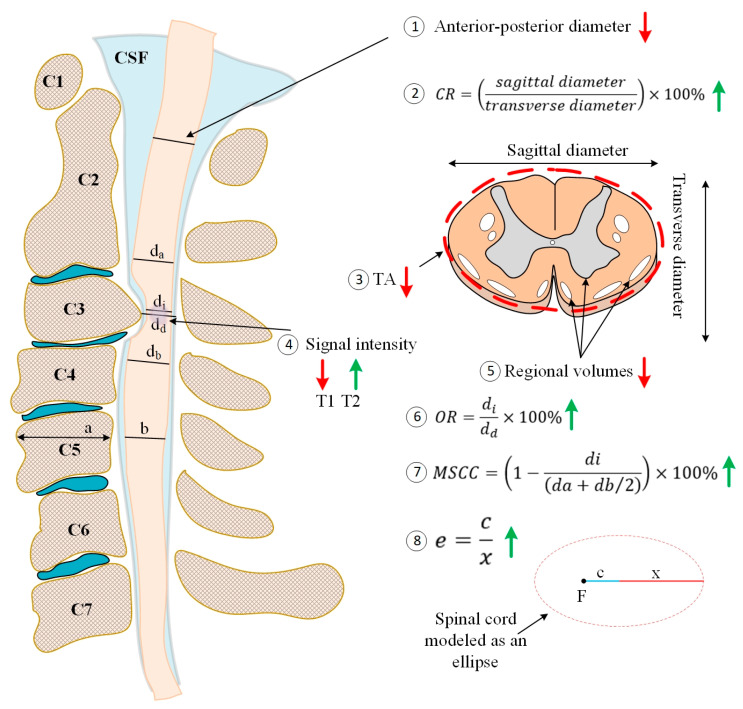
Macrostructural changes in the cervical spinal cord assessed using quantitative metrics calculated from T1- or T2-weighted MRI data. A vertical arrow next to each metric denotes whether the metric typically decreases (red) or increases (green) at the site of the spinal cord injury. Abbreviations: CSF: cerebrospinal fluid, CR: compression ratio, e: eccentricity, MSCC: maximum spinal canal compression, OR: occupation rate, TA: transverse area.

Magnetic resonance imaging (MRI) has been extensively used to qualitatively and quantitatively understand, diagnose, and monitor pathologies of the spine and recently also in the brain in CSM patients (Figure 1). MRI sequences, including T1-weighted (T1WI or T1) and T2WI (or T2) imaging sequences, have been extensively utilized in interrogating macro-level CSC tissue injury (Figure 2). However, they cannot typically detect minor abnormalities in tissues that appear otherwise normal on T1/T2 scans [10,11], making the diagnosis a challenge. Such microstructural CSC tissue injuries can occur at an earlier stage of disease progression [12] and have been reported using MRI methods, including diffusion-weighted imaging (DWI) [13], magnetization transfer (MT) imaging [14], and magnetic resonance spectroscopy (MRS) [12]. Furthermore, there is emerging evidence of compensatory functional reorganization in the brain [7,15], providing evidence of the impact of CSM on the CNS beyond the spinal cord. Such functional changes are primarily assessed using functional MRI (fMRI), a neuroimaging method sensitive to changes in neural tissue oxygenation [16]. While structural MRI has primarily interrogated the white matter (WM) pathology, the recent availability of fMRI provides an opportunity to examine functional changes in the gray matter (GM) of the spinal cord and the brain in CSM.

This review aims to present the utility of MRI methods in assessing the structural and functional changes in the CNS in CSM. The rest of this review is structured as follows. Section 2 briefly describes clinical metrics for evaluating CSM severity. Section 3 and Section 4 detail the MRI methods in assessing tissue injury at a macrostructural and microstructural level, respectively. Section 5 discusses the utility of fMRI in CSM research. Section 6 discusses challenges and solutions that hinder the clinical utility of MRI methods. Section 7 provides conclusions. It should be noted that the majority of the CSM research findings presented in this review pertain to the cervical spinal cord rather than the brain. Therefore, unless explicitly stated otherwise, it may be assumed that the context of the review pertains to the cervical spinal cord.

## 2. The Heterogeneity of CSM Poses a Diagnostic and Treatment Challenge

The natural history of CSM is poorly understood, and symptom manifestation among patients varies significantly [9]. Therefore, the current clinical assessment measures are not ideal for CSM’s overall assessment and evaluation. In other words, the choice of clinical metric(s) depends on the research question. For example, the Neck Disability Index (NDI) assesses how neck pain affects patients’ ability to manage their everyday life activities [17]. The Nurick grading system primarily assesses the lower extremity [18]. The modified Japanese Orthopedic Association (mJOA) score assesses upper and lower motor and sensory, including bladder dysfunction [19]. Despite the availability of several clinical metrics, developing an effective metric for CSM is still an active area of research. Researchers must have an adequate understanding to choose appropriate clinical metrics for their study.

## 3. Macrostructural Tissue Injury

This section describes MRI methods for quantifying macro-level tissue injury.

### 3.1. Compression of the Cervical Spinal Cord

CSC compression is one of the most common types of tissue injury, resulting from the spinal canal’s narrowing and spinal cord’s compression. Spinal cord compression in CSM can occur at a single or multiple vertebral or spinal levels. These pathologies are visible on a conventional T1 or T2 MRI scan. Studies have shown that the degree of spinal cord compression correlates with the clinical severity of CSM [3,20,21,22,23,24] and is often a significant factor in the surgical decision-making process [25].

The anterior-posterior diameter (APD) is calculated as the distance between the anterior and posterior ends of the spinal cord (Figure 2). APD is reduced in CSM [3,26,27,28,29,30] and can predict worsening mJOA scores, making it a useful clinical quantitative metric for assessing disease severity [26]. Occupation rate (OR) is calculated as the ratio of the sagittal diameter of the spinal cord and the sagittal diameter of the neural canal at the site of compression [29] (Figure 2). It is increased in CSM [31] and can be used as a guide to performing laminoplasty on CSM patients with ossification of the posterior longitudinal ligament [32]. APD and OR change with age, sex, and spinal column height [29]. The transverse area (TA) is calculated as the area the spinal cord occupies on an axial MRI slice (Figure 2). The TA, also called the spinal cord cross-sectional area, is typically reduced due to spinal cord compression in CSM [20,33].

**Figure 3:**
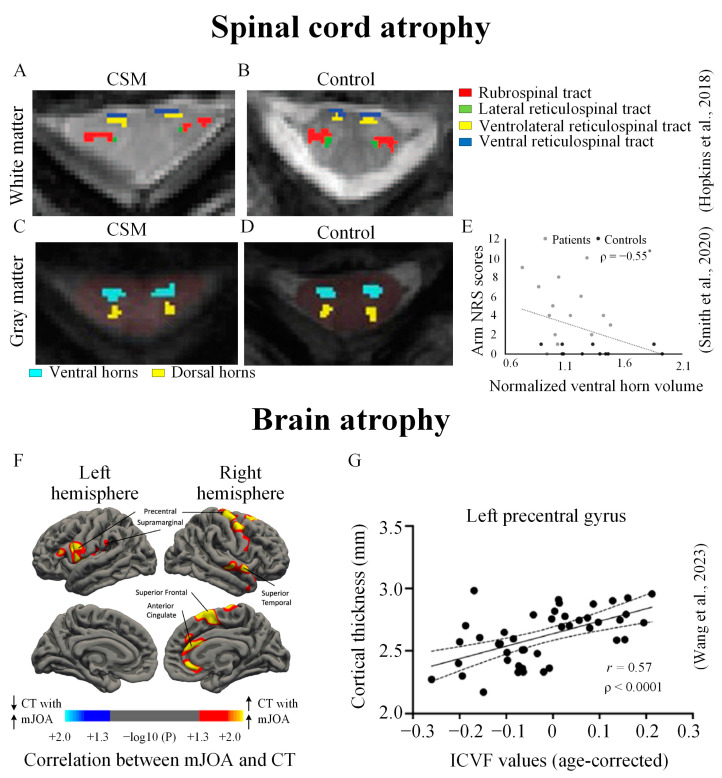
Brain and spinal cord atrophy in CSM. Spinal cord tract-specific white matter volume reduced in a (**A**) CSM patient compared to a (**B**) healthy control; reduced ventral horn volume in a (**C**) CSM patient compared to a (**D**) healthy control; (**E**) the reduced ventral horn volumes were negatively correlated with the arm Numeric Rating Scale (NRS) scores (*); (**F**) cortical regions demonstrating a strong association between the cortical thickness (CT) and mJOA score in a cohort of both CSM patients and healthy controls. Thresholding based on level of statistical significance (*p* < 0.05); (**G**) significant correlation between cortical thickness and intracellular volume fraction (ICVF, representing neurite density) values in the left precentral gyrus (adapted with permission from [5,6,26]).

Maximum spinal cord compression (MSCC) measures spinal cord compression with reference to the normal reference values above and below the site of compression [27] (Figure 2). MSCC provides a quantifiable and reliable assessment of spinal cord compression and has been applied in several CSM studies [20,25,34,35,36,37,38,39]. The compression ratio (CR), defined as the ratio between the sagittal diameter and transverse diameter (Figure 2), can also assess spinal cord compression [3]. The CR is reduced in CSM [20,33,40,41] and can assess disease severity [20]. A metric similar to the CR is the CSC eccentricity that measures the “roundness” of the spinal cord (Figure 2). In healthy conditions, the uncompressed spinal cord presents as close to a circle (the eccentricity of a circle is 0). It may increase in a compressed state [26] (see Figure 3A for an example of a compressed CSC).

Signal intensity aberrations of the spinal cord relative to a baseline reference can also be used as a quantitative metric to assess spinal cord injury [33,42]. Such signal intensity changes depend on the type of MRI contrast used. For example, abnormalities in the spinal cord manifest as areas with hyperintensity and hypointensity on T2 and T1 images, respectively. T2 hyperintense signal changes represent edema, inflammation, and changes in tissue water content. In contrast, hypointensity on T1 images is associated with more significant clinical impairment, such as permanent injury [43,44].

### 3.2. Atrophy of the Spinal Cord and Brain

The spinal cord descending tract volumes may be reduced in patients with CSM patients. Moreover, this white matter atrophy may predict disease severity (Figure 3A,B) [45]. In a recent study, spinal cord atrophy has also been found in gray matter volumes in CSM patients [5]. Specifically, the study found that the normalized ventral and dorsal gray matter volumes in the compression region were significantly lower in patients compared to controls (Figure 3C,D). Moreover, the gray matter volumes were associated with clinical metrics (Figure 3E).

There is emerging evidence that structural changes occur beyond the spinal cord in the brain [6,46,47,48,49]. For example, a recent study investigating the interrelationship between brains’ gray matter and subcortical white matter alterations in CSM patients reported that cortical thinning of sensorimotor and pain-related regions was associated with more severe clinical symptoms (Figure 3F,G) [6]. Another volume-based morphometry analysis study reported bilateral clusters of gray matter loss in the sensorimotor cortex and pulvinar nucleus [48]. Volume loss has also been reported in other brain areas, including the supplementary motor area and cerebellum [46]. Combined, the above studies suggest that region-specific volume loss or atrophy of the CSC and brain can potentially be used as biomarkers reflecting the pathology of CSM.

## 4. Microstructural Tissue Injury

This section describes MRI methods with the potential to assess tissue injury at a microstructural level.

### 4.1. Exploiting Water Movement to Assess White Matter Tissue Injury

**Figure 4:**
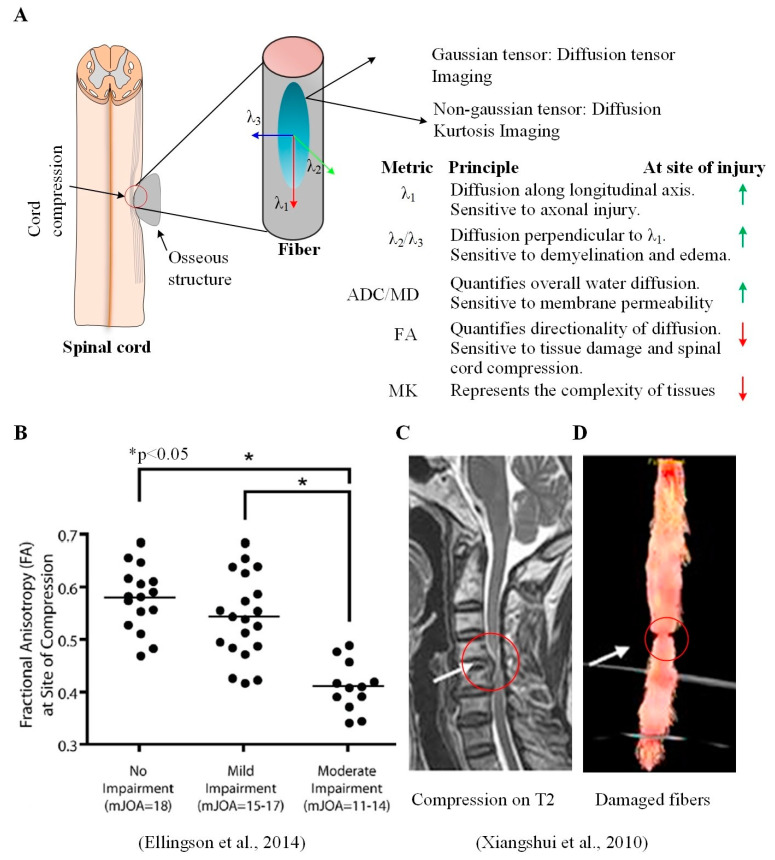
Diffusion tensor imaging (DTI) and fiber tractography (FT) can assess microstructural tissue changes. (**A**) The diffusion of water molecules in the tissues is assumed to be an ellipsoid and is mathematically modeled as a tensor matrix. In an isotropic medium (for example, CSF), the ellipsoid has a spherical shape, whereas it elongates and forms a beam in tissue structures such as axons. DTI metrics can be calculated using individual entries of the tensor matrix. These metrics typically decrease (vertical red arrow) or increase (vertical green arrow) at the site of tissue injury; (**B**) FA at the site of spinal cord compression can distinguish CSM severity; (**C**) cord compression on T2 image of a CSM patient at the C5–C6 level with subtle hyperintensity and corresponding (**D**) fiber tractography map showing a significant distortion at the compression site (adapted with permission from [13,52]). Abbreviations: ADC: apparent diffusion coefficient, FA: fractional anisotropy, MD: mean diffusivity, MK: mean kurtosis. λ_i_ represents eigen value of the diffusion tensor matrix.

Diffusion tensor imaging (DTI), a subset of DWI, exploits that water molecules diffuse differently along the tissues depending on factors such as the type of tissues, structural integrity, and presence of barriers, etc. A diffusion tensor model can mathematically describe the rate and direction of water diffusion at the level of a voxel [50] (Figure 4A). Specifically, the magnitude of water diffusion along the primary axis (or direction) of nerve fibers is quantified by the longitudinal (or axial) diffusion (LD). LD is sensitive to tissue injury and typically increases in the CSC in CSM patients [51,52,53]. Similarly, the water diffusion perpendicular to the primary axis of the nerve fibers is quantified by the radial diffusivity (RD), which quantifies the degree of restriction to water molecules due to membranes and other structures. RD represents demyelination and edema [54,55] and is typically increased in the CSC in CSM patients [13,51,52,53,56].

A weighted sum of diffusivities along the three axes of the fibers is given by the apparent diffusion coefficient (ADC), alternatively called the mean diffusivity (MD), which quantifies direction-invariant water diffusion rate [57] and is also typically increased in the CSC in CSM patients [51,52,53,58,59,60,61,62,63,64,65,66] and is positively correlated with age [60].

Another DTI metric, fractional anisotropy (FA), describes the anisotropy of molecular diffusion at the level of a voxel [67]. FA is sensitive to tissue injury [68,69] and is typically decreased in the CSC in CSM patients [51,52,58,59,60,61,62,70]. DTI can assess spinal cord microstructure at the level of the spinal column. For example, a DTI study reported that FA was significantly lower in the lateral and posterior spinal columns. At the same time, MD, axial diffusivity, and radial diffusivity were significantly higher in the anterior, lateral, and posterior spinal column in CSM patients than in healthy controls [51].

Diffusion kurtosis imaging (DKI) is an extension of DTI with a non-gaussian water diffusion profile. It provides information complementary to ADC and FA metrics [71]. For example, DKI mean kurtosis represents tissue complexity [70,72,73,74]. DKI, compared to DTI, can potentially more accurately quantify microstructural tissue changes in the brain [75,76] and has shown promise for evaluating brain and spinal cord disorders in vivo [70,77].

Fiber tractography (FT) is a graphical variant of DTI used for assessing microstructural tissue changes via post-processing DTI tensor solution and constructing a white matter fiber map of the brain [78] and spinal cord [52,79]. FT may provide greater accuracy in diagnosing lesions in the CSC in CSM patients than in T2 images alone [61,62]. Figure 4D shows an example of an FT map of the CSC of a representative CSM patient [52]. This study demonstrated that ADC, FA, and diffusivities along each axis were significantly increased, decreased, and increased, respectively, in CSM patients compared to healthy controls. In contrast, the fiber maps were helpful in visually verifying changes in the compressed cord.

There has been a recent increase in the use of model-based DWI methods in CSM, such as diffusion basis spectrum imaging (DBSI) [80] and neurite orientation and dispersion density imaging (NODDI) [81]. DBSI is an extension of DTI to provide a more comprehensive evaluation of the microstructural changes via multiple tensors, providing quantitative metrics beyond what DTI may offer [82,83]. NODDI can distinguish between changes in the axons, the extracellular fluid and matrix, and the presence of glial cells. This can provide a more accurate assessment of the extent and nature of the microstructural changes occurring in the spinal cord and brain and is increasingly being used in CSM studies [6,84,85].

### 4.2. Reduced Myelin Content Is Associated with Tissue Injury

**Figure 5:**
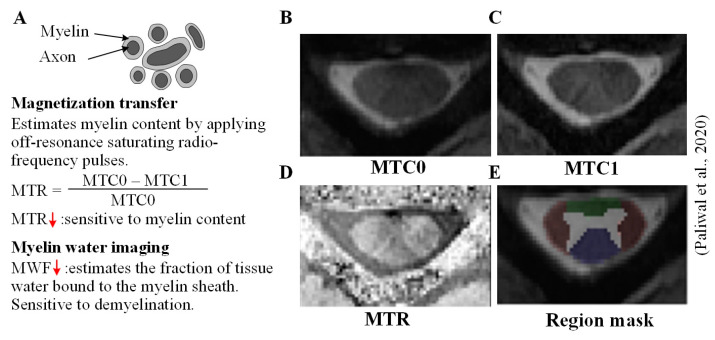
Estimating myelin content in CSM. (**A**) Neuronal fibers constitute axons wrapped heavily in a myelin sheath. The myelin content typically undergoes deterioration in CSM and can be estimated using magnetization transfer (MT) imaging and myelin water imaging; (**B**) an example of an axial slice of the cervical spinal cord without MT pulse (MTC0), (**C**) with MT pulse MTC1, (**D**) MTR, and (**E**) overlaid regional masks (adapted with permission from [94]).

MT imaging [86] and myelin water imaging (MWI) [4] have been used to estimate myelin content which is typically reduced in the CSC in CSM patients and is associated with functional impairment scores [4]. MT employs off-resonance saturating radio-frequency pulses, causing the protons bound to macromolecules to be “activated.” These bound protons then transfer their saturation to nearby “free” proton pools, effectively saturating the MRI signal [87]. Using the voxel data in the saturated and unsaturated state, a ratiometric metric, i.e., MT ratio (MTR) sensitive to myelin content, can be used (Figure 5A). While MTR is associated with the degree of axonal and myelin damage in the brain [88], MT has also shown potential in studying spinal cord pathologies [89,90,91,92,93]. Although MT has been employed in the spinal cord for more than twenty years, this MRI method has recently been used to study its diagnostic value [45,94,95,96] and ability to predict recovery [94] in the spinal cord in CSM patients. When combined with other techniques, MT imaging can provide complementary useful diagnostic information. For example, a multimodal DTI and MT imaging study assessed tract-specific microstructural information on the spinal cord and their clinical correlations in CSM patients [96].

MWI estimates myelin content by estimating the fraction of tissue water bound to the myelin sheath. This is accomplished by fitting the T2 relaxation curve to a multi-exponential model and identifying the fraction of the signal with a T2 parameter [97]. This MW fraction (MWF) can be applied to assess myelination in the spinal cord [98]. MWF is approximately 50% higher in the spinal cord than normal brain white matter and varies along the length of the spinal cord [99] while decreasing with age [100]. While numerous spinal cord MWF studies on multiple sclerosis (MS) can be found [10], there is limited literature on CSM. A notable CSM study on the spinal cord provided in-vivo anatomical evidence of demyelination related to conduction deficits [4].

### 4.3. Exploiting Metabolite Concentrations to Study Tissue Injury

MRS interrogates disease-related metabolic changes before the manifestation of the abnormality of the tissue at the cellular or organ level is detectable in a routine clinical MRI examination [12]. This spectroscopic method quantifies either the absolute or relative concentrations of specific small molecules of interest, also called metabolites, within a single large voxel. For example, N-acetyl aspartate (NAA) is primarily contained in neurons [101], so it can be used as a measure of neural density and as a biomarker for neural integrity [102,103]. Choline (Cho) can potentially be a biomarker for tracking neural membrane degradation [101]. Creatine (Cr) is relatively stable as it is related to global metabolic activity and can be used as a control [104]. The presence of lactate (Lac) peak in an MRS spectrum indicates anaerobic metabolism that could be related to inflammation or ischemia [103,105]. Glutamate-Glutamine (Glx) can be used as a biomarker for assessing changes in glutamatergic pathways. For example, a decreased Glx indicated abnormalities in the glutamatergic pathways in the cervical spinal cord in patients with MS but without spinal cord atrophy [106]. Some of these metabolites are age-dependent [107]. They are statistically significantly different in different anatomical areas; for example, the cervical spinal cord and brain stem [108].

MRS has been used primarily in the human brain [109], and the literature on MRS in the spinal cord is limited. Nevertheless, several studies have demonstrated the potential of MRS in assessing microstructural tissue changes in the CSC in CSM [12,103,110,111,112,113]. Combining MRS with other MRI methods has shown clinical utility. For example, combining DTI with single-voxel MRS was found to help predict neurological impairments in CSM patients [112]. Specifically, significant correlations were observed between DTI CSC fiber tract density and FA, MD, Cho/NAA, and FA and Cho/NAA. Moreover, DTI metrics and MRS metabolites at the C2 level (Cho/NAA) at the site of spinal cord compression were significantly correlated with mJOA scores. In another study, NAA/Cr and Cho/NAA ratios in the CSC could predict mJOA score after surgery, demonstrating their predictive utility [111].

## 5. Using Functional Changes as a Proxy to Study Tissue Injury

Functional MRI (fMRI) allows noninvasive assessment of the brain [114] and spinal cord [115], providing functional information complementary to the structural information described in Section 3 and Section 4. These functional changes are detected at the voxel level to create a blood oxygenation-level dependent (BOLD) signal [114]. These time-series data can be processed, analyzed, and used to interpret functional activation and connectivity changes in the CNS in CSM. Recently, spinal cord fMRI has been gaining momentum and has been successfully used in a wide array of experimental paradigms, including motor [116,117,118] and sensory [119,120,121] tasks and in resting state studies [122,123,124].

**Figure 6:**
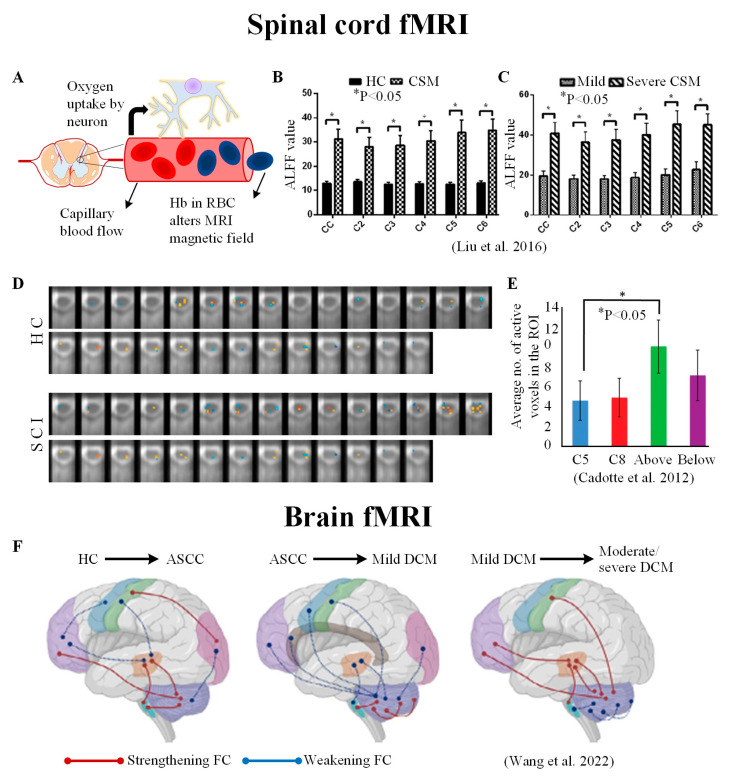
Functional MRI of the central nervous system in CSM and spinal cord injury (SCI). (**A**) Underlying hemodynamic mechanisms for fMRI. Blood supplies oxygen and energy to active neurons, which then deoxygenate the red blood cells (RBCs) and alters the local MRI magnetic field. (**B**,**C**) Resting-state measurements of frequency fluctuations (ALFF) in CSM patients have shown to be associated with increasing myelopathic severity; (**D**,**E**) when compared to healthy controls (HCs), SCI patients were found to have more average number of active voxels above the region of injury; (**F**) functional reorganization in the brain during different stages of myelopathy. (Figures adapted with permission from [7,122,125].) Abbreviations: ASCC: asymptomatic spinal cord compression, DCM: degenerative cervical myelopathy, FC: functional connectivity, Hb: deoxygenated hemoglobin.

One emerging application of fMRI is to study the pathophysiology of CSM in the CSC [122,125] and brain [7] (Figure 6). Recent evidence suggests that CSM pathophysiology involves neuronal plasticity, or the ability of the brain to reorganize itself, which has the potential to occur early in CSM [126,127]. For example, studies on the brain suggest an increased functional activity in the cerebellum, which could be responsible for the minimization of movement errors, movement timing, and fine motor movement in CSM [126,128]. Additionally, decreased functional connectivity between the somatosensory cortex and other motor areas indicates neuronal impairment within the brain [127]. Furthermore, post-surgical recovery involves decreased functional activity in the brain’s motor areas, suggesting improvement in motor learning [126,129]. The brain’s visual cortex is also impacted by CSM, as studies indicated a decreased functional connectivity in the visual cortex that improved after surgery [15,128].

In addition to brain fMRI, resting-state spinal cord fMRI has also been applied to CSM. After analyzing the amplitude of low-frequency fluctuations (ALFF) of BOLD signals in the spinal cord, it was found that the BOLD signal increased with the symptomatic severity in CSM patients [122]. This finding corroborated with findings in the brain, as ALFF signals in the precentral gyrus were found to have increased in correlation with JOA measurements in CSM patients, suggesting a requirement for an increased compensatory functional reorganization [130] (Figure 6B,C). This increased neuronal activity within the spinal cord of CSM patients supports evidence for neuronal plasticity within the CNS, which plays a crucial role in maintaining normal bodily functions and coping after a specific part of the CNS has been damaged. The ability of fMRI to interrogate neuronal plasticity can help further understand how it plays a role in CSM and other neurological injuries and pathologies, such as spinal cord injury and MS. For example, studies have reported detecting functional activity below the site of CSC injury during both sensory and motor stimulation [16,125,131,132] and altered patterns of activation in the ventral and dorsal horns [16,131] and intraspinal connectivity levels [125].

The use of fMRI in CSM is relatively new, but it continues to increase in popularity as imaging methodologies improve. In the future, it could be an integral part of learning about the underlying neuronal mechanisms within the CNS and observing fundamental changes that could lead to successful treatments.

## 6. Discussion

The current paper reviewed research that suggests a potential to investigate tissue injury in CSM patients via quantitatively measuring structural and functional changes in the central nervous system. While MRI-based neuroimaging research for CSM has progressed by leaps and bounds in the past two decades, several challenges still exist that are a hurdle to clinical translation, especially in interrogating the spinal cord. The first, and perhaps the biggest challenge, is the anatomy of the spinal cord. The spinal cord has a small cross-sectional area compared to the surrounding structures. These structures consist of significantly different tissues from the spinal cord with different magnetic susceptibility. This significant difference in susceptibility may cause distortions in the magnetic field, resulting in a distorted image and reduced intensity, thus making it challenging to capture small structural details of the spinal cord [133]. Imaging in the transverse axis (axial slices) results in fewer distortions [134]. However, it can increase the acquisition time if a large field of view is required [133].

Second, MRI metrics of the spinal cord are affected by artifacts arising from cardiac and respiratory motion and cerebrospinal fluid pulsation [79,135]. These artifacts can be minimized using cardiac pulse gating to improve signal quality [136]. Other techniques to reduce signal artifacts include parallel imaging and single-shot echoplanar imaging. However, these require longer scanning time, and because CSM patients with severe symptoms typically cannot endure additional scanning time in the MRI suite, scan acquisition time is still a problem for these patients [68]. In addition to hardware-based corrections, corrections in the post-processing steps can be applied to reduce artifacts from the different sources described above [133].

Third, MRI metrics depend on age as described above. Therefore, MRI measurements should be compared in patients with age-matched control participants. When age matching is not feasible, age may be adjusted in multivariate analysis [137].

Fourth, multimodal studies provide more information than is available from a single MRI method. The number of multimodal studies has been increasing recently. It should be considered in designing future studies to assess insults to the spinal cord and brain in CSM patients accurately.

Fifth, it is complex and time taking to accurately segment the spinal cord from its surrounding tissues in healthy individuals compared to the brain. It becomes challenging in lesioned areas such as regions of compression. The tissue segmentation becomes even more difficult at the level of the individual spinal cord tract. As a result, studies often perform manual segmentation by one or more experienced radiologists. Even then, they typically include the gray and white matter without discriminating between them. Performing a manual spinal cord segmentation is not only laborious but is also prone to bias and its accuracy depends on the operator’s skill. Recently, the Spinal Cord Toolbox (SCT) [138] has been increasingly utilized to help automate the segmentation process at the level of tracts, using a probabilistic approach. Several studies have utilized it [81,84,94,95,96]. Another utility of the SCT is using atlases, e.g., PAM50, to perform group-level analysis [139]. Therefore, efforts are needed to develop such automation software.

Sixth, standardization is needed, especially at the acquisition stage, to increase the reproducibility of experimental findings. Efforts in this direction are being made. For example, the spine generic protocol provides guidance on using either of the three most used 3T MRI machines from Philips, GE, and Siemens [140]. Other efforts include vendor-neutral sequences [141,142] and open-source pulse sequences [143]. Such standardization allows the comparison of results across multiple sites and researchers, thus helping synergize collaborative efforts toward translating CSM research into the clinic.

Finally, although the number of studies examining the brain in the context of CSM is increasing, these investigations typically focus on the brain and spinal cord separately. This may be due to the additional time required to perform simultaneous scans of both regions of the central nervous system. Further analysis that compares the structural and functional impairments in both the brain and spinal cord could advance our comprehension of the underlying pathophysiology of CSM.

Several promising MRI methods that have not yet been applied to CSM, have been applied to other pathologies of the central nervous system. For example, R2* MRI, which quantifies the iron content in tissues and lesions [144], has been applied to multiple sclerosis in the human brain [145] and could potentially be applied to CSM patients. Another promising MRI method is susceptibility-weighted imaging, an MRI sequence sensitive to calcium content [146], which is an element thought to have an essential role in neurodegenerative disorders such as axonal loss in multiple sclerosis [147]. These emerging techniques can potentially help further our understanding of the pathology of CSM beyond what is possible from existing MRI methods and help accelerate the progress toward developing reliable, quantifiable biomarkers for CSM that are translatable to the clinical environment.

While the current paper focused on MRI techniques, it should be noted that several non-MRI imaging modalities exist that are clinically used or have shown potential in the diagnosis and management of CSM. One such example is X-ray imaging in 2D [148] or 3D (i.e., computed tomography) [149] that provides anatomical images allowing clinicians to visualize degenerated tissues such as osteophytes, damaged discs, and ossifications in the ligaments in high contrast. This imaging modality is frequently used in a clinical setting, is relatively fast, and is particularly useful in surgical planning. Furthermore, such imaging methods may be helpful in discriminating CSM from inflammatory diseases such as myelitis and sarcoidosis, which affect soft tissues.

Recently, electrophysiology studies have also shown usefulness in CSM diagnosis and management. For example, nerve conduction investigation helps rule out peripheral nerve abnormalities in myelopathic patients [150]. Moreover, the use of somatosensory evoked potentials [4,59,151] and motor evoked potentials [152,153,154] in the patient population have shown potential in serving as a diagnostic biomarker and application in intraoperative monitoring [155]. Electrophysiological studies are typically time consuming and may be uncomfortable to the patients, which limits their practicality. However, they are evolving and hold promise for the future [150]. In summary, diagnosing and managing CSM requires a combination of imaging methods and clinical metrics. MRI is the preferred imaging method, but other techniques such as CT and electrophysiology, may also be useful, depending on the individual patient’s clinical presentation and available resources.

## 7. Conclusions

MRI provides a promising suite of methods for investigating the pathophysiology of CSM and for monitoring the effects of therapeutic interventions using structural and functional neuroimaging of the brain and spinal cord. With advances, these methods will likely become an increasingly important tool for diagnosing and managing CSM. However, several challenges must be understood and worked on to help in the clinical translation of these methods.

## Data Availability

Not applicable.
